# Supplementary material for: Baicalin Protects Broilers against Avian Coronavirus Infection via Regulating Respiratory Tract Microbiota and Amino Acid Metabolism
Source: Int J Mol Sci. 2024 Feb 9;25(4):2109. doi: 10.3390/ijms25042109 (PMC10888704; doi:10.3390/ijms25042109)
Supplement: Supplementary file 1 [file ijms-25-02109-s001.zip › ijms-2746978-supplementary.pdf]

Supplementary Figures

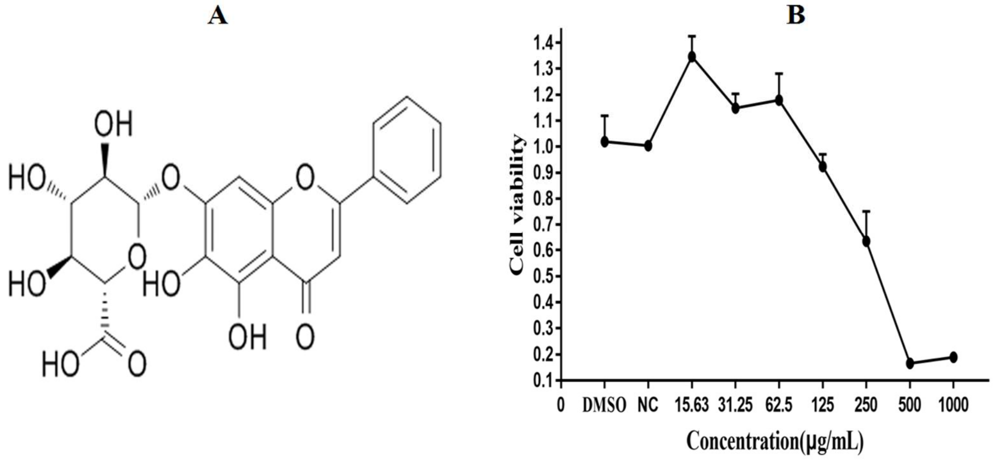

Supplemental Figure S1. A represents the molecular structure of Bai. **B** indicates the relative cell viability of the CEK cells in the presence of the Bai.

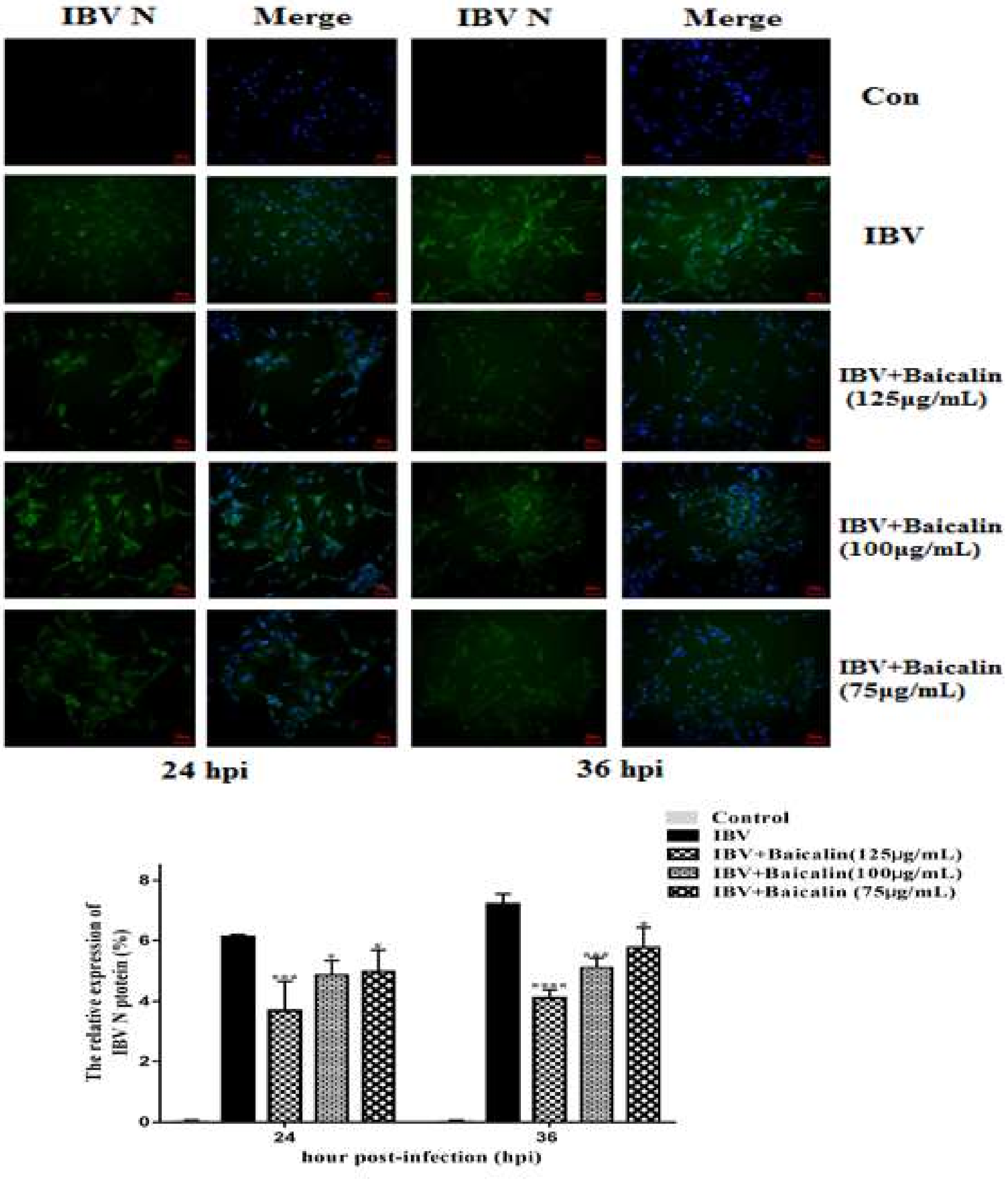

Supplemental Figure S2. The effect of Bai on IBV fluorescence expression in IBV-infected CEK cells. The green indicated the IBV N fluorescence, and blue indicated the DAPI. The signal fluorescence were analysed by Image-J software.

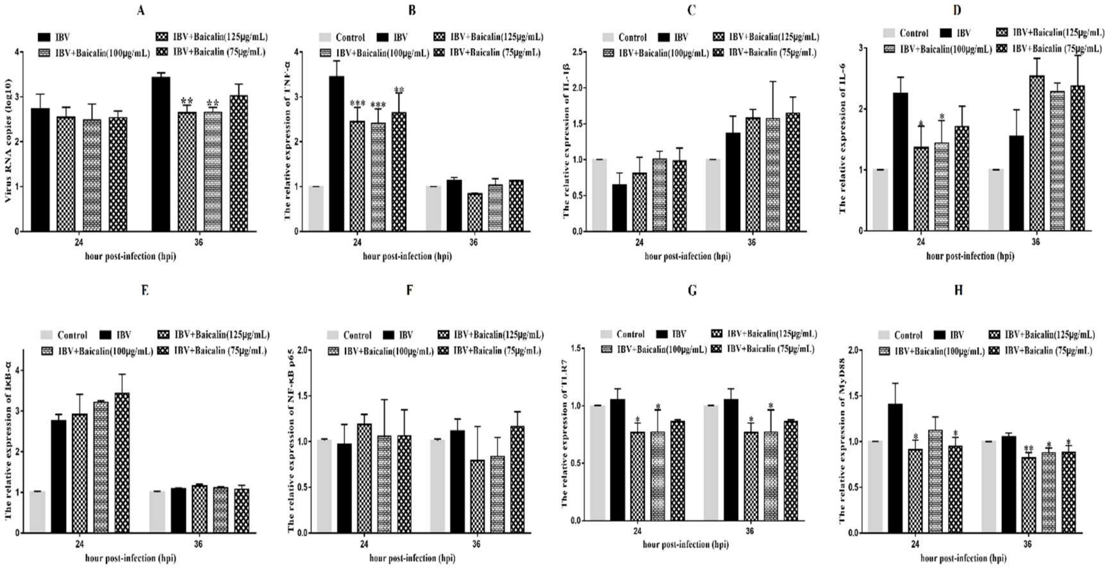

Supplemental Figure S3. A - **D** represent the expression of IBV copies, TNF-α, IL-1β and IL-6 at 24 and 36 hpi. **E** - **H** represent the expression of IκB-α, NF-κB, TLR7 and MyD88 at 24 and 36 hpi.

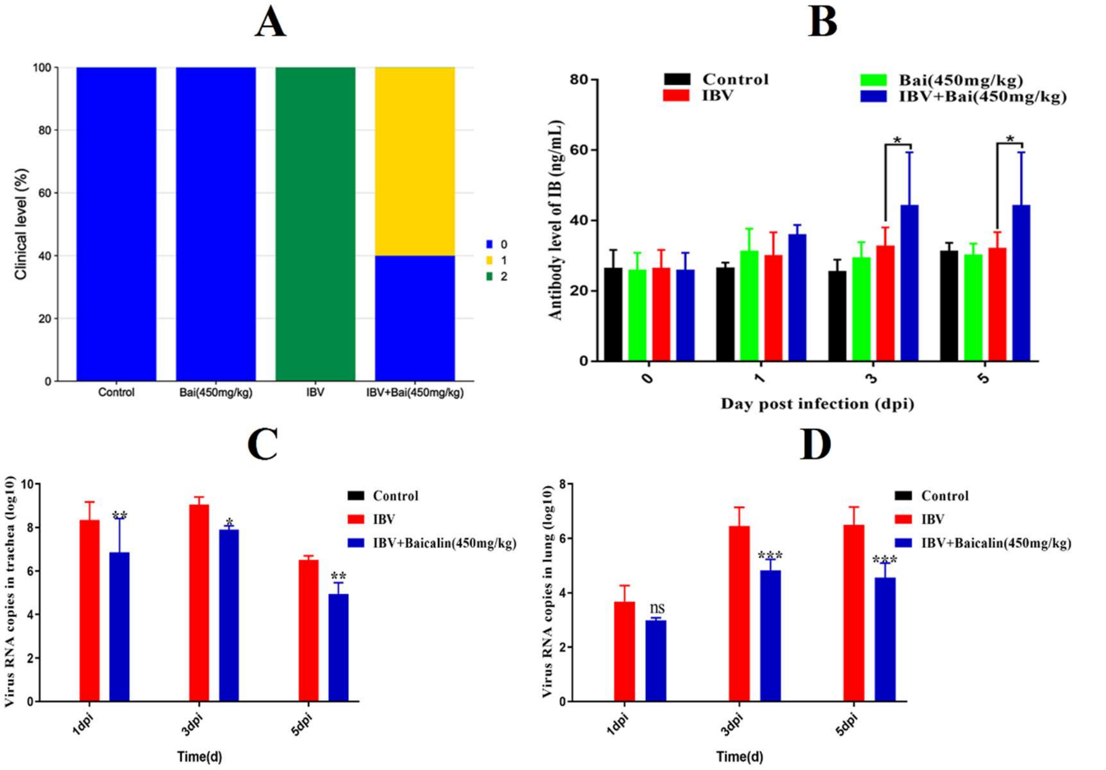

Supplemental Figure S4. Bai relieved the decreasing of the growth performance and immune response in broilers. **A** represents the clinical level. Note: 2 represents the significant clinical symptoms, 0 represents that there were no significant clinical symptoms, 1 was between 0 and 2. **B** represents anti-IBV antibody level. **C** and **D** represent virus loads in the trachea and lung, respectively.

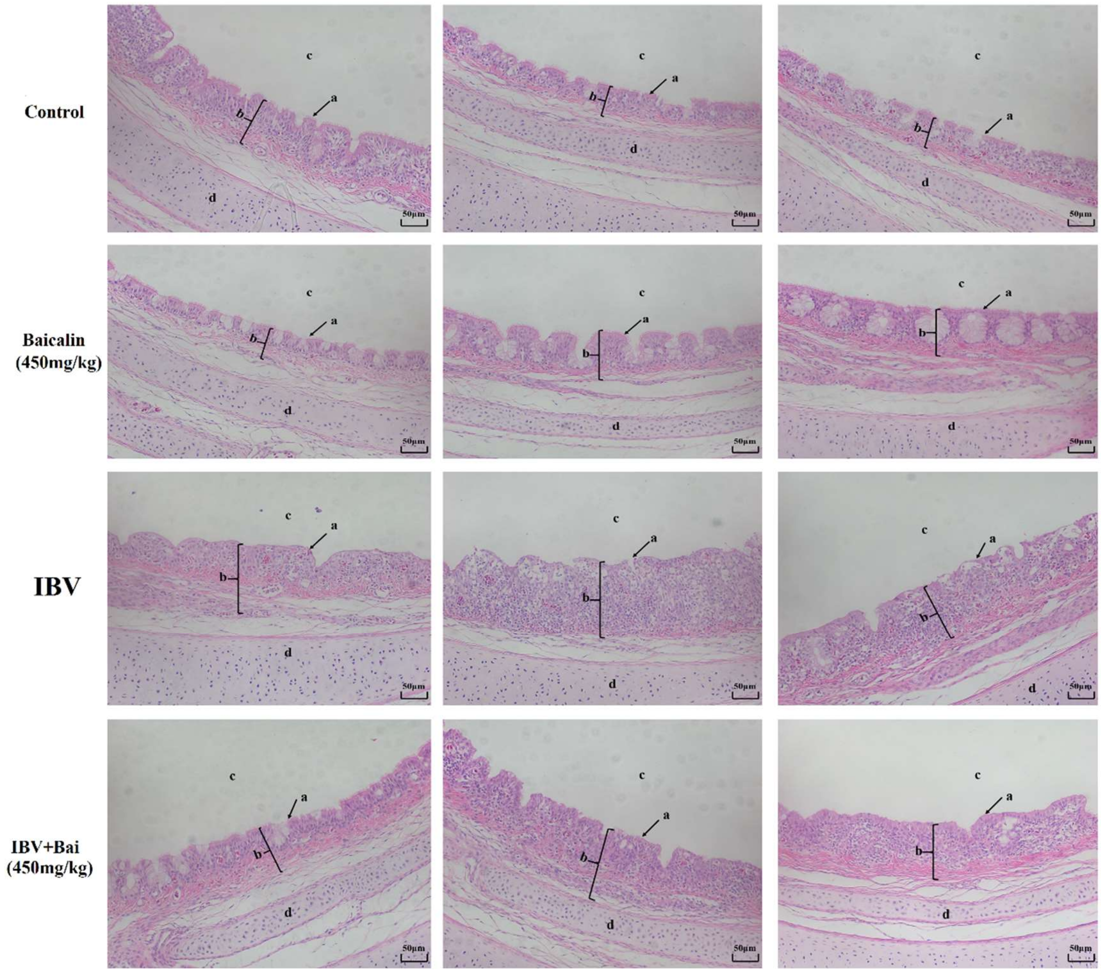

Supplemental Figure S5. The tracheal histopathological changes in broilers at 1, 3 and 5 dpi (magnification, 200×). **A** indicates cilia; **b** indicates trachea mucosa; **c** indicates trachea lumen; **d** indicates trachea cartilage.

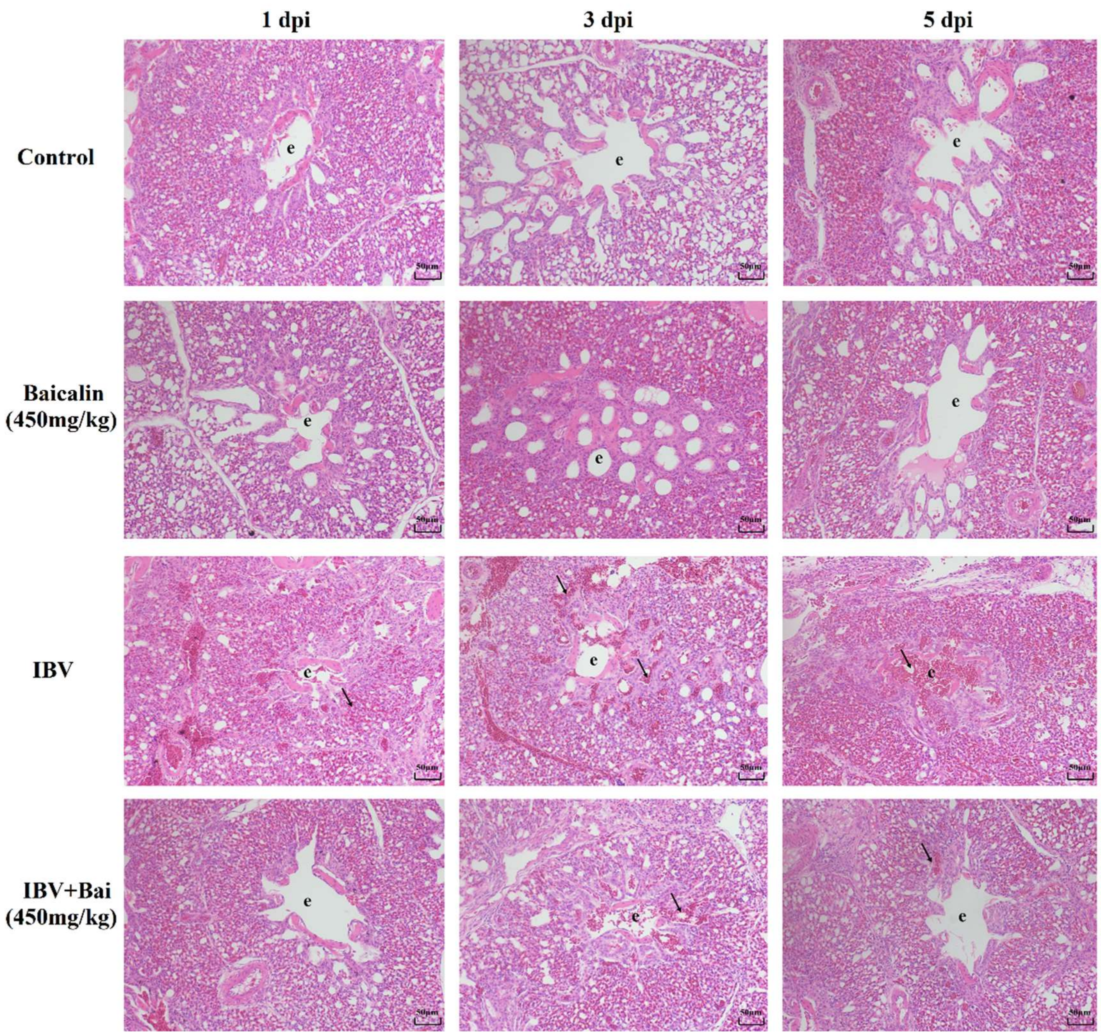

Supplemental Figure S6. The histopathological changes of lung in broilers (magnification, 200×), **e** indicates 4arabronchial lumen.

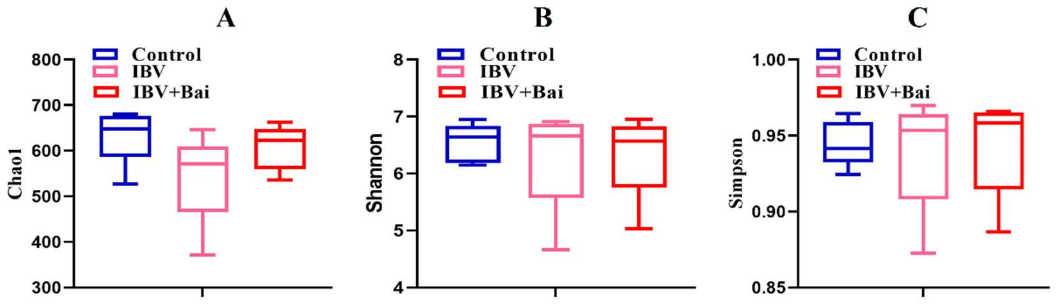

Supplemental Figure S7. A, **B**, **C** were the indices of Chao1, Shannon and Simpson, respectively.

Supplementary methods

2.1. ELISA for anti-IBV antibody

The IBV-specific antibody in serum was quantified by commercial ELISA kit (Jiubang, Fujian, China) according to the manufacturer's instruction, which exhibits a detection range spanning from 5 ng/mL to 160 ng/mL, demonstrating a remarkable sensitivity of 1.0 ng/mL. Briefly, the samples were added to a 96-well microplate coated with antigens, followed by adding 100 μL antibody conjugated with Streptavidin–HRP, before then being incubated for 1 h at RT. Furthermore, substrate solution was added to each well. Then, the reaction was stopped and the optical density was detected at 450 nm.

*2.2. Histopathological detection and immunohistochemistry analysis (**IHC**)*

Fixed tracheal tissues were embedded in paraffin and then sectioned at 5 μm thickness, then stained with haematoxylin and eosin. In addition, the slide was incubated with 3% hydrogen peroxide for 15 min at RT after being dewaxed and dehydrated. Then, the slide was washed 3 times with Tris Buffered Saline (**TBS**) and incubated with primary antibody (1:500) at 4 ºC in a humidified chamber overnight after being blocked with 10% normal goat serum for 30 min at 37 ºC. Further, tissue sections were incubated with secondary antibody for 30 min at 37 ºC and washed 3 times with TBST for 5 min. Slides were incubated with diaminobenzidine (**DAB**) for 5 min, washed with distilled water, counter-stained with hematoxylin for 3 min, dehydrated and observed under a light microscope (Olympus, Japan).

2.3. Scanning Electron Microscopy analysis (SEM)

The tracheal tissues fixed in 2.5% glutaraldehyde were blocked into 1% OsO_4 for 1-2 h at RT after being rinsed with phosphate buffer 3 times. After that, they were washed with phosphate buffer 3 times. Next, the tissues were dehydrated followed by gradient alcohol and isoamyl acetate for 15 min. The samples were dried with a Critical Point Dryer and sputter-coated with gold for 30s. Lastly, the tissues were observed and had images taken with a scanning electron microscope (SEM, HITACHI, Japan).

*2.4. Real-time quantitative polymerase chain reaction (**RT-**qPCR)*

Total RNA from CEK cells and equiponderant tissues were extracted using TRIzol reagent (Takara, Dalian, China). The RNA was reversed to cDNA using PrimeScript RT reagent kit with gDNA Eraser (Takara, Dalian, China) based on the manufacturer's instructions. The gene expressions of related factors were determined using a 7500 real-time PCR system (Applied Biosystems, Foster, CA, USA) with SYBR®Premix Ex Taq™ II (Takara, Dalian, China). The mRNA expression was calculated with the $2^{-\Delta\Delta}$ threshold cycle (**CT**) method, with normalisation relative to β-actin. The primer sequences are listed in **Table 2**.

*2.5. Western Blot (**WB**) analysis*

Total proteins were extracted from CEK cell and tracheal tissue with a RIPA Lysis Buffer (Beyotime, Shanghai, China). Equivalent amounts of protein were separated by SDS-PAGE, followed by transferal onto polyvinylidene-difluoride (**PVDF**) membranes by electroblotting. The membranes were reacted with the primary antibody at 4 °C overnight after being blocked with QuickBlock Blocking Buffer, followed by washing thrice with TBST. Then, membranes were incubated with a goat anti-mouse/rabbit IgG HRP secondary antibody for 1 h and washed with PBST thrice. Finally, protein bands were visualised using chemiluminescence reagent, and the gray value of the corresponding protein was analysed by Image-J software. The detailed antibody information used in this work was showed in **Table 3**.

2.6. Respiratory microbial structure changes by 16S rDNA analysis

16S rDNA sequencing analysis was performed by BIOTREE Biological Technology Co., Ltd., Shanghai, China. In brief, bacterial genomic DNA samples were extracted from broilers' tracheas according to the E.Z.N.A.® Stool DNA Kit (D4015, Omega, Inc., USA). The

hypervariable region V3-V4 of the bacterial 16S rRNA gene was amplified using the primer pairs 341F (5'-CCTACGGGNGGCWGCAG-3') and 805R (5'-GACTACHVGGG TATCTAATCC-3') in the polymerase chain reaction (**PCR**) thermocycler ABI GeneAmp 9700 (ABI, CA, USA). The amplified genes were filtered and cleaned up using AMPure XT beads (Beckman Coulter Genomics, Danvers, MA, USA). The amplicon pools were subjected to sequencing by employing the Illumina NovaSeq PE250 platform. Both the Library Quantification Kit for Illumina (Kapa Biosciences, Woburn, MA, USA) and Agilent 2100 Bioanalyzer (Agilent, USA) were employed to evaluate the length and amount of the amplification library, respectively. Specific sequences were converted into equivalent random sequences, and the resulting sequences' alpha diversity and beta diversity were computed. The SILVA (version 132) classifier normalized the feature abundance based on the relative proportion of the gene sequences. The Shannon and Simpson indices in the samples were computed using QIIME2. The alpha diversity of the intestinal microbiota was calculated using the index of Shannon, Simpson, Chao, and Operational Taxonomic Units (OTUs).

2.7. Analysis and identification of metabolites

Freeze-dried tracheas of 25 mg was placed into an Eppendorf tube, and 500 μL extract solution was added. Then the samples were was homogenized for 4 min at 40 Hz in a rotating ball mill (JXFSTPRP-24, Shanghai Jingxin Technology Co., Ltd., China), and then for a period of 5 min in 3 repetitive cycles in ice water using an ultrasonic generator (PS-60AL, Shenzhen Redbond Electronics Co. Ltd., China). The samples were subsequently incubated for 1 h at -40 ºC and centrifuged (Heraeus Fresco 17, Thermo Fisher Scientific, USA) for 20 min at 5000 rpm. The supernatant was added with an internal standard made of 2-methylvaleric acid (25 mg/L; Dr. Ehrenstorfer, Germany) and 0.1 mL of 50% sulfuric acid (Sinopharm, China). Following that, it was subjected to -20°C incubation for 30 min. The incubated supernatant was transferred into a new glass vial non-target metabolism analysis, which adopted the test methods of Shanghai Biotree Biotech Co. Ltd. (Shanghai, China), using the gas chromatography-mass spectrometry (GC-MS; GC2030-QP2020 NX, Shimadzu, Japan) technique. The HP-FFAP (30 m × 250 μm × 0.25 μm; J&W Scientific, Folsom, CA, USA) capillary column of the gas chromatography system (Agilent, Folsom, CA, USA) was filled with one μL supernatant. At an average flow rate of 1 mL/min, helium was employed as the transport gas. The initial operating temperature was kept at 80°C for 1 min. It was then heated to 200°C at 10°C/min and maintained at 200°C for 5 min. A further heating to 240°C at 40°C/min was introduced, and the operating temperature was kept at 240°C for another min. Temperatures for the injection front, transfer line, quad, and ion source were set at 240°C, 240°C, 150°C and 200°C respectively. The MS test was carried out with an electron ionization energy of -70 eV. The MS data with a m/z spectrum ranging from 33-150 were collected in the Scan/SIM mode following a 3.5 min solvent delay.
